# Is there an association between pelvic organ prolapse and oxidative stress? A systematic review

**DOI:** 10.1371/journal.pone.0271467

**Published:** 2022-08-04

**Authors:** Dominik Habes, Jan Kestranek, Jaroslav Stranik, Marian Kacerovsky, Jiri Spacek

**Affiliations:** 1 Department of Obstetrics and Gynecology, Faculty of Medicine, University Hospital Hradec Kralove, Charles University, Hradec Kralove, Czech Republic; 2 Biomedical Research Center, University Hospital Hradec Kralove, Hradec Kralove, Czech Republic; University College London, UNITED KINGDOM

## Abstract

**Introduction and hypothesis:**

The pathophysiology of pelvic organ prolapse (POP) has not been fully elucidated, although accumulating evidence suggests that oxidative stress is involved. The present systematic review comprehensively discusses this topic.

**Methods:**

The PubMed/Medline, Scopus, and Web of Science databases were searched for relevant studies published up to May 2021. This systematic review was registered in the PROSPERO database (registration number CRD42021242240). Two independent researchers screened and selected articles that fulfilled predefined inclusion criteria, performed a quality assessment, and extracted the relevant data. Of 901 original articles retrieved, 8 fulfilled the selection criteria and were included in the review.

**Results:**

Elevated levels of markers of oxidative stress, such as advanced glycation end products, hydroxynonenal and hydroxydeoxyguanosine, were found in various parts of the pelvic floor of patients with POP. Accordingly, the levels of glutathione peroxidase and superoxide dismutase, known as major antioxidant enzymes, were reduced, compared to those in healthy controls. Levels of two other markers (mitofusin 2 and nuclear factor erythroid derived 2) also support hypotheses suggesting the involvement of oxidative stress in POP.

**Conclusions:**

In the literature available, an association between oxidative stress and pelvic organ prolapse was confirmed.

## Introduction

Pelvic organ prolapse (POP) is a common disease, especially among postmenopausal women. The lifelong prevalence of POP based on a vaginal examination is up to 50% of all women [1]. According to some sources, surgical treatment is required in 11% of cases [2]. The etiology of POP has not been fully elucidated; however, its origin is believed to be multifactorial.

According to the life span model proposed by DeLancey et al., risk factors for POP can be divided into 3 groups: predisposing factors (genetic predisposition and nutritional factors); inciting factors (vaginal delivery and parity); and intervening factors (occupational lifting, obesity, chronic cough, chronic steroid use, and aging) [2, 3].

Based on presence of these risk factors, several hypotheses have been proposed to explain the origins of POP. The most often cited explanation is based on the influence of vaginal birth-related pelvic floor trauma (e.g., levator ani avulsion, endopelvic fascia tear or pudendal nerve damage) [4]. Avulsion of the levator ani during childbirth has been confirmed using magnetic resonance imaging, as well as its association with POP [5]. Pudendal nerve damage has been reported to be an underlying cause of levator ani muscle atrophy [6].

To date, however, the time lag in the development of POP—years or even decades after these events—has not been fully explained. A possible explanation is the effect of oxidative stress (OS)—as a characteristic feature of aging—on the endopelvic fascia once damaged by delivery-associated trauma. There is accumulating evidence that OS is involved in the pathophysiology of POP. However, this evidence is from studies that assessed only selected markers of OS in various parts of the female genital tract. The present review aims to provide comprehensive information about the role of OS in POP.

## Material and methods

### Protocol and registration

The literature search was performed in accordance with the Preferred Reporting Items for Systematic Reviews and Meta-Analyses (i.e., “PRISMA”) checklist/methodology. It is presented in S1 Appendix [7]. This systematic review was registered in the PROSPERO database (registration number CRD42021242240).

### Search strategy

The literature search was performed using the PubMed/Medline, Scopus, and Web of Science databases. Only the key points are described here, the entire search strategy is presented in S2 Appendix. The terms used in the search included “pelvic organ prolapse,” “oxidative stress,” “reactive oxygen species,” “advanced glycation end products” “8-hydroxydeoxyguanosine,” “4-hydroxy-2-nonenal,” “isoprostane,” “superoxide dismutase,” “glutathione peroxidase,” and “Mitofusin 2”.

### Eligibility criteria

Criteria for study inclusion were as follows: women with confirmed POP of any severity, without any restrictions to age, race, socioeconomic status, menopausal status, parity, or delivery method. The aim was to determine whether the POP was associated with changes in the concentrations of OS markers in body tissues or fluids. There were no limitations to year of publication, and all available studies published up to May 2021 were searched. All study designs, except for letters, comments, non-systematic reviews, and practice guidelines, were considered to be potentially eligible. Studies involving animal models and in vitro studies using cell cultures, cell lines, and stem cells were excluded. Furthermore, studies with participants < 18 years of age were also excluded.

### Study selection

All studies retrieved in the literature search were classified using the Rayyan app [8]. Duplicates were removed, and all remaining studies were screened according to title and abstract. Only those studies that potentially met the inclusion criteria were selected for full-text review, of which only those that fulfilled all inclusion criteria were included. All studies were screened by 2 independent researchers (DH and JS). In case of disagreement, a third reviewer and senior member of the staff (JK) rechecked the studies and made the final decision.

### Quality assessment and analysis

Quality assessment was performed using the Joanna Briggs Institute critical Appraisal tool for Analytical Cross Sectional Studies (JBI Appraisal tool) [9], and the evaluation was performed by 2 independent researchers (DH and JS). In case of disagreement, a third reviewer (JK) rechecked the study and made the final decision. Results of the quality assesment are summarized in “Table 1”.

**Table 1:** Selected data of markers of oxidative stress in patients with POP.

Author	Study design, number of subjects	POPQ (case)	Diagnosis (control)	OS Marker	Method	Outcome	Risk of bias
Li, B.S. et al	cross-sectional	3 cohorts, POP^a^ 2, 3 and 4	other benign gynecological diseases	GPx1 ^c^	IHC^k^	decreased levels of GPx1^c^	5/8
	n (case): 30						
	n (control): 20						
Chen, H. Y. et al	cross-sectional	POP^a^ 2–4	benign gynecological diseases	Mfn2^d^	qRTPCR^l^ + westernblot	elevated expression of Mfn2^d^	6/8
	n (case): 37						
	n (control): 23						
Fang, G. et al	cross-sectional	2 cohorts, POP^a^ 2, POP^a^ 3 + 4	CIN ^b^ II-III	8-OHdG^e^, 4-HNE^f^, SOD^g^, GPx1^c^	8-OHdG^e^ + 4-HNE^f^ IHC^k^, SOD^g^ + GPx1^c^ qRTPCR^l^ + westernblot	elevated levels of 8-OHdG^e^ and 4-HNE^f^ in POP^a^ 3+4 group, increased expression and decreased levels of SOD^g^ and GPx1^c^ in POP^a^ 3 + 4 group	5/8
	n (case): 40						
	n (control): 20						
Kim, E. J. et al	cross-sectional	3 cohorts, POP^a^ 2, 3 and 4	benign gynecological diseases	8-OHdG^e^, 4-HNE^f^	IHC^k^ + TUNEL ASSAY	elevated levels of 8-OHdG^e^, 4-HNE^f^ in POP^a^ group	5/8
	n (case): 26						
	n (control): 29						
Vetuschi, A. et al	cross-sectional	POP^a^ 3–4	benign gynecological disorders	AGE^h^, RAGE^i^	IHC^k^ + westernblot	elevated levels of AGE^h^, RAGE^i^ decreased or absent in POP^a^ group	4/8
	n (case): 20						
	n (control): 10						
Lin, T. et al	cross-sectional	POP^a^ 3–4	benign gynecological diseases	** **GPx3^c^, Nrf 2^j^	IHC^k^ + westernblot	Levels of GPx3^c^ and Nrf 2^j^ decreased	5/8
	n (case): 35						
	n (control): 35						
Liu, C. et al	cross-sectional	POP^a^ 2–4	benign indications, including CIN^b^	8-OHdG^e^, 4-HNE^f^	IHC^k^	elevated levels of 8-OHdG^e^ and 4-HNE^f^	6/8
	n (case): 20						
	n (control): 20						
Chen, Y. et al	cross-sectional	POP^a^ 3–4	CIN ^b^, benign ovarian lesions	AGE^h^, RAGE^i^	IHC^k^ + westernblot	elevated levels of AGE^h^, no difference for RAGE^i^	5/8
	n (case): 44						
	n (control): 46						

^a^ Pelvic organ prolapse

^b^ cervical intraepitelial neoplasia

^c^ glutathione peroxidase

^d^ mitofusin 2

^e^ hydroxydeoxyguanosin

^f^ hydroxynonenal

^g^ superoxide dismutase

^h^ advanced glycation end-products

^i^ advanced glycation end-products receptors

^j^ Nuclear factor (erytroid derived) 2

^k^ immunohistochemistry

^l^ quantitative real time polymerase chain reaction

### Data extraction

The following data were extracted from the included studies: study design, number of cases and controls, origin of tissue sample, methods of evaluation of POP-Q score in the case group, selection criteria for the control group, OS marker and detection method used, and major outcome(s).

## Results

### Description of studies

**Fig 1:**
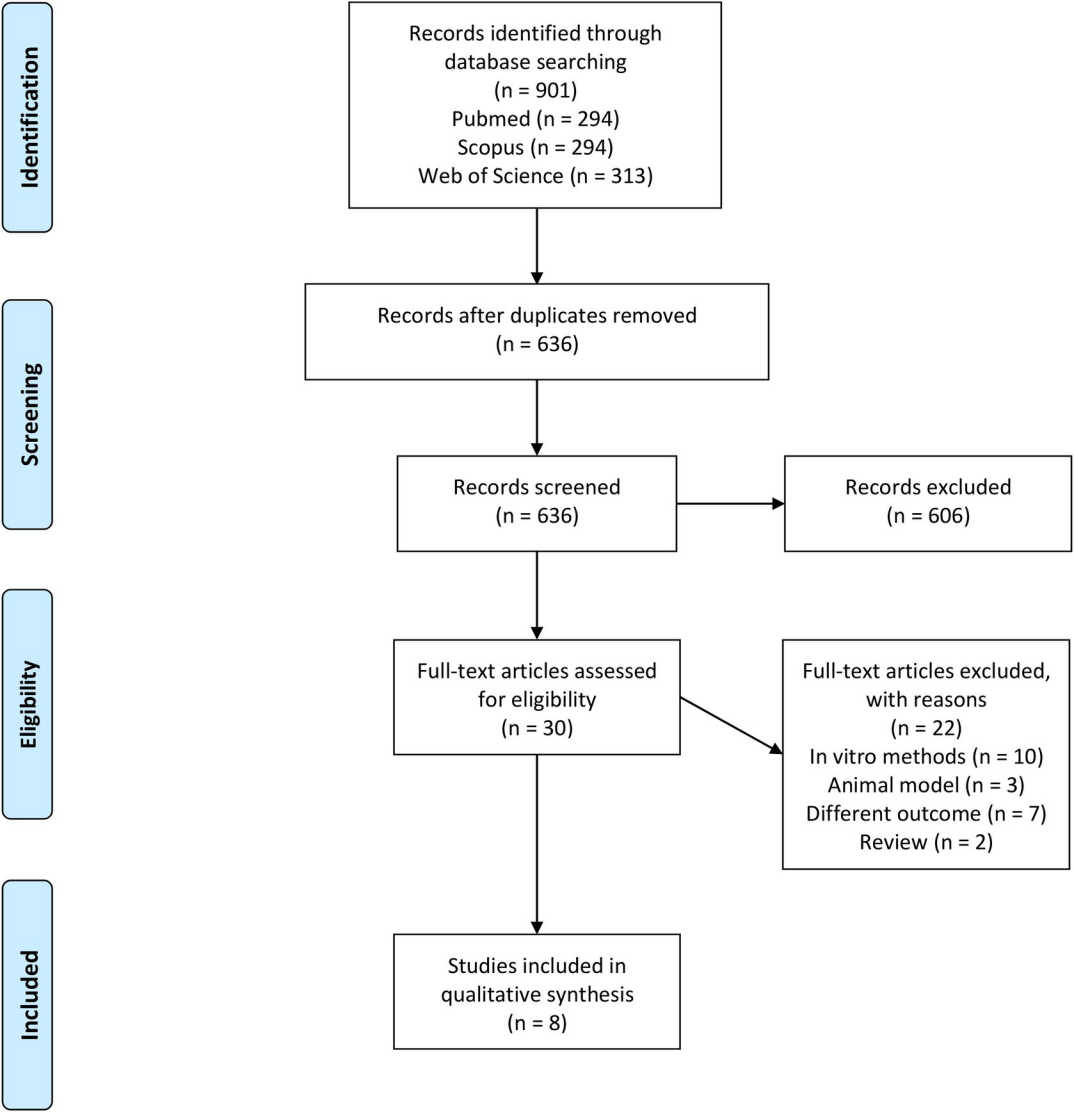
PRISMA flow diagram [7].

The initial search retrieved 901 studies. Using the Rayyan app, 265 duplicates were removed and the remaining 636 were screened by title and abstract, of which 606 were excluded because they did not fulfill all inclusion criteria. 30 studies were selected for full-text review, of which 22 were excluded, ultimately leaving 8 for inclusion. Reasons for exclusion were as follows: use of in vitro techniques (n = 10), use of animal models (n = 3), different type of presented outcome than OS marker changes in women with POP (n = 7) and non-systematic reviews (n = 2). A flow-diagram of the study selection process is presented in “Fig 1”.

The main characteristics of the selected studies are summarized in “Tables 1 and 2”; all 8 studies were cross-sectional in design. Overall, data from 455 women, including 252 cases, were extracted. When all the parameters of selected studies were evaluated, it was found that the performance of meta-analysis was not possible owing to the presence of heterogeneity in the described markers, sampling sites and used analytical methods.

**Table 2:** Markers of oxidative stress in pelvic organ prolapse tissues.

OS Marker	Method	Outcome in POP:	Sample origin	Author(s)
**8-OHdG** ^**a**^	IHC^j^	elevated levels	cardinal ligament	Fang, G. et al
	IHC^j^	elevated levels	uterosacral ligament	Kim, E. J. et al, Liu, C et al.
**4-HNE** ^**b**^	IHC^j^	elevated levels	uterosacral ligament	Kim, E. J. et al, Liu, C et al.
	IHC^j^	elevated levels	cardinal ligament	Fang, G. et al
** AGE** ^**c**^	IHC^j^ + westernblot	elevated levels	vaginal wall	Vetuschi, A. et al., Chen, Y. et al
**RAGE** ^**d**^	IHC^j^ + westernblot	decreased or absent levels	vaginal wall	Vetuschi, A. et al
	IHC^j^ + westernblot	unchanged levels	vaginal wall	Chen, Y. et al
**GPx1** ^**e**^	IHC^j^	decreased levels	pubocervical fascia	Li, B.S. et al
	qRTPCR^k^ + westernblot	increased expression and decreased levels	cardinal ligament	Fang, G. et al
** GPx3** ^**f**^	IHC^j^ + westernblot	decreased levels	vaginal wall	Lin, T. et al
**SOD** ^**g**^	qRTPCR^k^ + westernblot	increased expression and decreased levels	cardinal ligament	Fang, G. et al
**Mfn2** ^**h**^	qRTPCR^k^ + westernblot	elevated expression	uterosacral ligament	Chen, H. Y. et al
**Nrf 2** ^**i**^	IHC^j^ + westernblot	decreased levels	vaginal wall	Lin, T. et al

^a^ hydroxydeoxyguanosin

^b^ hydroxynonenal

^c^ advanced glycation end-products

^d^ advanced glycation end-products receptors

^e^ glutathione peroxidase 1

^f^ glutathione peroxidase 3

^g^ superoxide dismutase

^h^ mitofusin 2

^i^ Nuclear factor (erytroid derived) 2

^j^ immunohistochemistry

^k^ quantitative real time polymerase chain reaction

### OS markers

OS markers were described in various parts of the pelvic floor, including the uterosacral ligaments [10–12], cardinal ligaments [13], different parts of the vaginal wall (anterior wall [14, 15] and fornix [16]), and pubocervical fascia [17].

Evidence of either elevated OS marker levels or decreased levels of major antioxidant enzymes was reported in all 8 selected studies (see “Tables 1 and 2”). Advanced glycation end products (AGEs) [14, 16], hydroxynonenal (HNE) [11–13], and hydroxydeoxyguanosine (OHdG) [11–13] were used as markers of OS. Gpx [13, 15, 17] and superoxide dismutase (SOD) [13] were considered to be major antioxidant enzymes. Furthermore, two studies used mitofusin 2 (Mfn2) expression [10] and levels of nuclear factor erythroid derived 2 (Nrf2) [15] as OS markers.

HNE and OHdG were used in 3 studies [11–13], all of which reported elevated levels of both markers in the case group compared to those in controls. AGE and receptor for advanced glycation end products (RAGEs) were evaluated in 2 studies [14, 16], with elevated levels of AGE reported in both articles. Outcomes in the RAGE analysis differed. Vetuschi et al. [14] stated that RAGE in the case group were decreased or absent, but Chen et al. [16] reported no difference between the case and control group.

Gpx levels were investigated in 3 studies [13, 15, 17], all of which described decreased levels (according to immunohistochemistry/Western blot). In 1 study [13], increased expression of Gpx (according to quantitative real-time polymerase chain reaction [qRTPCR]) was confirmed.

SOD was used in 1 study [13], in which increased expression but decreased levels was reported. Mfn2 was used in 1 study [10], in which elevated expression levels were reported. Nrf2 was analyzed in 1 study, in which expression was lower in POP samples than in the control group.

In summary, apart from conflicting results in RAGE analysis, all selected markers of oxidative stress were elevated and all selected antioxidant enzymes were decreased despite their increased expression.

### Analytical methods

Immunohistochemistry was used to assess Gpx [15, 17], OHdG, HNE [11–13], AGE, RAGE [14, 16] and Nrf2 [15] levels in 7 studies. Two studies used semi-quantitative analysis of immunohistochemical results, in both cases were blinded. The remaining 5 studies used quantitative software from 2 different manufactures.

Western blotting was used in 5 studies for AGE, RAGE [14, 16], Gpx [13, 15], SOD [13], Mfn2 [10], and Nrf2 [15] detection. These 5 studies used 3 different analytical software packages to analyze the results, although 1 study failed to report the methods used. qRTPCR was used in 2 studies to evaluate gene expression of Gpx, SOD [13], and Mfn 2 [10].

## Discussion

The aim of this systematic review was to confirm elevated levels of OS markers in tissues obtained from patients with POP. OS markers were found in various segments of the pelvic floor, including the vaginal wall, and the cardinal and uterosacral ligaments. All 8 of the included studies supported the hypothesis of increased OS in POP-affected tissues. The following molecules were selected as markers of OS: AGEs, RAGE, HNE, OHdG, Gpx, SOD, Nrf2 and Mfn2. Levels of these markers were determined by immunohistochemical and Western blot methods. However, comparability of the semiquantitative evaluation of the results was diminished due to differences in the methodologies used in individual studies.

OS is characterized as an imbalance between oxidants and antioxidants in favor of oxidants. Oxidants include reactive oxygen and nitrogen species (ROS and RNS, respectively) [18]. As a result of elevated levels of ROS and RNS, physiological cell signaling and functions are disrupted by altered proteins, DNA, and lipids. This leads to a reduction in the number of fibroblasts and collagen fibrils. The result of long-term impaired cellular function is gradual worsening of damage to the pelvic floor, which represents one of the basic mechanisms of aging [12, 18].

OS markers are involved in responses to OS at various levels; as such, the levels of these markers fluctuate accordingly. In general, the concentration of molecules generated as by-products of OS (i.e., AGE, HNE, OHdG) or proteins mediating the reaction (i.e., Mfn2), will increase. Conversely, proteins that function in response to excessive OS will be more consumed by excessive activity (RAGE, Gpx, SOD and Nrf 2); as such, despite increased expression, their levels will decrease. This is probably due to the increased consumption of these antioxidant enzymes. Most of the results published in the included studies support this statement. Only the results for RAGE were not consistent.

AGEs are a by-product of the non-enzymatic reaction between reducing sugars and lipids or, more often, proteins and, under physiological conditions, are involved in cell signaling [18]. Several in vitro studies have demonstrated that the overexpression of AGEs is associated with decreased expression of collagen, and multiple possible molecular mechanisms have been proposed [16, 19]. Numerous other changes to the pelvic floor have also been described, especially to the endopelvic fascia, which have been attributed to the development of POP. Among other mechanisms, remodeling of the extracellular matrix by degradation of collagen fibers by matrix metalloproteinases (MMPs) [14, 20, 21] has been proposed. The association between AGE and MMP expression has also been demonstrated, but only in vitro [19]. Unfortunately, however, AGEs are the only OS markers of which the mechanism of action is relatively known.

HNE is a lipid oxidation end product [18] and is probably involved in cell signaling as a second messenger in OS-induced apoptosis [22]; however, the current knowledge base is limited. Current data were obtained by investigating cells other than POP-affected fibroblasts, such as liver cancer cells [18]. Generalization of these mechanisms, therefore, should be made with caution.

OHdG is a product of a radical reaction involving guanosine. In general, OHdG can disrupt DNA transcription; however, the impact of these changes on the development of POP remains unclear.

Gpx and SOD are antioxidant defense enzymes that ensure OS reduction by inactivation of ROS/RNS. Multiple isoenzymes have been described. Two studies focused on Gpx 1, and one on Gpx 3. SOD is suspected to play a role in protecting DNA/RNA against OS by interacting with proteins involved in DNA repair [13].

Mfn2 is a transmembrane protein in mitochondria with different roles in cellular metabolism. Among others, it is responsible for cellular reaction to OS [23]. However, its involvement in multiple cellular processes limits the interpretation of the results. Thus, it is difficult to know whether increases in its expression are due to higher OS or other factors, such as age or menopausal status.

Nrf2 is a transcriptional factor and a regulator of antioxidant defense. It regulates approximately 250 different genes, including those of antioxidant enzymes. Due to its wide range of effects, it is involved in several other processes, including aging. Therefore, it is debatable to what extent altered levels of Nrf 2 can be attributed to POP and to other factors, such as age.

This review focuses only on the description of OS in ex vivo acquired tissues. But one important OS marker (isoprostanes) had not been studied this way, the only available data were obtained using in vitro methods. Choy et al. [24] focused on determining whether or not there is elevated isoprostane´s production in cardinal ligaments of POP patients compared to those from healthy controls. The study conclusion aligns with our conclusions, elevated markers of OS had been found in POP group. Furthermore, similar results have been proven in urine samples of POP patients in comparison to healthy controls, which provides an alternative way of evaluation of the disease status in vivo. Knowledge of the quality of the fibrous component of the pelvic floor could potentially play a role in determining adequate therapy. However, further research is needed.

To our knowledge, only two studies addressed the topic of antioxidant supplementation as a preventive precaution of POP. Unfortunately both used animal models [25, 26]. Both studies show promising results. But given the complex pathophysiology of POP and the major physiological differences between these two mammals and humans regarding delivery, we don´t believe that any conclusion can be drawn from these results.

On the other hand, some studies have already addressed other potential ways in which OS affects POP. With use of in vitro methods, a positive correlation between mechanical stress and elevated levels of OS was confirmed [27]. It is believed, that mechanical stress in general is one of the triggers leading to enhancing OS [28]. And in case of POP, excessive mechanical stress on individual parts of the suspension apparatus cannot be disputed. Once the OS levels rise, further interference with collagen metabolism is provided by a pathway using AGE as described earlier [16, 19]. This theory offers a possible explanation, why heavy mechanical load (occupational lifting etc.) acts as a risk factor for developing POP. To observe the effects of limiting the mechanical stress placed on the suspense apparatus, a study is conducted by our team, that observes changes in OS markers after implanting a polypropylene mesh in treatment of POP patients.

The strength of this review is its focus on the quantification of the results of both Western blot and immunohistochemical evaluation of putative OS markers. In contrast, a limitation is, undoubtedly, the absence of a formal meta-analysis, which was not performed due to the lack of comparable data. Another limitation is the focus purely on westernblot and immunohistochemical studies. We are aware of the existence of proteomic and transcriptomic studies focused on the same topic. However, their non-inclusion in the study is in accordance with the inclusion criteria.

In conclusion, in the literature available, an association between oxidative stress and pelvic organ prolapse was confirmed.

## Supporting information

S1 Appendix Prisma checklist. (PDF)

S2 Appendix Search formula. (PDF)
