# Supplementary material for: Is there an association between pelvic organ prolapse and oxidative stress? A systematic review
Source: PLoS One. 2022 Aug 4;17(8):e0271467. doi: 10.1371/journal.pone.0271467 (PMC9352098; doi:10.1371/journal.pone.0271467)
Supplement: S2 Appendix — (PDF) [file pone.0271467.s002.pdf]

S1 Appendix 1 – Search formula for Pubmed, Scopus and Web of Science

PUBMED:

("POP"[All Fields] OR ("pelvic organ prolapse"[MeSH Terms] OR ("pelvic"[All Fields] AND "organ"[All Fields] AND "prolapse"[All Fields]) OR "pelvic organ prolapse"[All Fields]) OR ("prolapse"[MeSH Terms] OR "prolapse"[All Fields] OR "prolapses"[All Fields] OR "prolapsed"[All Fields] OR "prolapsing"[All Fields]) OR ("pelvic organ prolapse"[MeSH Terms] OR ("pelvic"[All Fields] AND "organ"[All Fields] AND "prolapse"[All Fields]) OR "pelvic organ prolapse"[All Fields] OR ("vaginal"[All Fields] AND "vault"[All Fields] AND "prolapse"[All Fields]) OR "vaginal vault prolapse"[All Fields]) OR ("uterine prolapse"[MeSH Terms] OR ("uterine"[All Fields] AND "prolapse"[All Fields]) OR "uterine prolapse"[All Fields] OR ("descensus"[All Fields] AND "uteri"[All Fields]) OR "descensus uteri"[All Fields]) OR "metroptosis"[All Fields] OR (("pelvic floor"[MeSH Terms] OR ("pelvic"[All Fields] AND "floor"[All Fields]) OR "pelvic floor"[All Fields]) AND ("abnormalities"[MeSH Subheading] OR "abnormalities"[All Fields] OR "defects"[All Fields] OR "defect"[All Fields] OR "defect s"[All Fields] OR "defected"[All Fields] OR "defective"[All Fields] OR "defectively"[All Fields] OR "defectives"[All Fields])) OR ("cystocele"[MeSH Terms] OR "cystocele"[All Fields] OR "cystoceles"[All Fields] OR "cystocoele"[All Fields] OR "cystocoeles"[All Fields]) OR ("cystocele"[MeSH Terms] OR "cystocele"[All Fields] OR "cystoceles"[All Fields] OR "cystocoele"[All Fields] OR "cystocoeles"[All Fields]) OR ("rectocele"[MeSH Terms] OR "rectocele"[All Fields] OR "rectoceles"[All Fields] OR "rectocoele"[All Fields] OR "rectocoeles"[All Fields]) OR ("rectocele"[MeSH Terms] OR "rectocele"[All Fields] OR "rectoceles"[All Fields] OR "rectocoele"[All Fields] OR "rectocoeles"[All Fields]) OR "uretrocele"[All Fields] OR (("anterior"[All Fields] OR "anteriores"[All Fields] OR "anteriorization"[All Fields] OR "anteriorized"[All Fields] OR "anteriors"[All Fields]) AND ("compartment"[All Fields] OR "compartment s"[All Fields] OR "compartments"[All Fields]) AND ("abnormalities"[MeSH Subheading] OR "abnormalities"[All Fields] OR "defects"[All Fields] OR "defect"[All Fields] OR "defect s"[All Fields] OR "defected"[All Fields] OR "defective"[All Fields] OR "defectively"[All Fields] OR "defectives"[All Fields])) OR (("middle"[All Fields] OR "middles"[All Fields]) AND ("compartment"[All Fields] OR "compartment s"[All Fields] OR "compartments"[All Fields]) AND ("abnormalities"[MeSH Subheading] OR "abnormalities"[All Fields] OR "defects"[All Fields] OR "defect"[All Fields] OR "defect s"[All Fields] OR "defected"[All Fields] OR "defective"[All Fields] OR "defectively"[All Fields] OR "defectives"[All Fields])) OR (("posterior"[All Fields] OR "posteriors"[All Fields]) AND ("compartment"[All Fields] OR "compartment s"[All Fields] OR "compartments"[All Fields]) AND ("abnormalities"[MeSH Subheading] OR "abnormalities"[All Fields] OR "defects"[All Fields] OR "defect"[All Fields] OR "defect s"[All Fields] OR "defected"[All Fields] OR "defective"[All Fields] OR "defectively"[All Fields] OR "defectives"[All Fields])) OR (("urogenital system"[MeSH Terms] OR ("urogenital"[All Fields] AND "system"[All Fields]) OR "urogenital system"[All Fields] OR "genitourinary"[All Fields]) AND ("prolapse"[MeSH Terms] OR "prolapse"[All Fields] OR "prolapses"[All Fields] OR "prolapsed"[All Fields] OR "prolapsing"[All Fields]))) AND ("oxidative stress"[MeSH Terms] OR ("oxidative"[All Fields] AND "stress"[All Fields]) OR "oxidative stress"[All Fields] OR ("oxidative stress"[MeSH Terms] OR ("oxidative"[All Fields] AND "stress"[All Fields]) OR "oxidative stress"[All Fields] OR ("oxidative"[All Fields] AND "damage"[All Fields]) OR "oxidative

damage"[All Fields]) OR ("reactive oxygen species"[MeSH Terms] OR ("reactive"[All Fields] AND "oxygen"[All Fields] AND "species"[All Fields]) OR "reactive oxygen species"[All Fields]) OR ("react oxyg species apex"[Journal] OR "ros"[All Fields]) OR ("glycation end products, advanced"[MeSH Terms] OR ("glycation"[All Fields] AND "end"[All Fields] AND "products"[All Fields] AND "advanced"[All Fields]) OR "advanced glycation end products"[All Fields] OR ("advanced"[All Fields] AND "glycation"[All Fields] AND "endproducts"[All Fields]) OR "advanced glycation endproducts"[All Fields]) OR ("8 hydroxy 2 deoxyguanosine"[MeSH Terms] OR "8 hydroxy 2 deoxyguanosine"[All Fields] OR "8 hydroxydeoxyguanosine"[All Fields]) OR ("8 hydroxy 2 deoxyguanosine"[MeSH Terms] OR "8 hydroxy 2 deoxyguanosine"[All Fields] OR "8 oxodg"[All Fields]) OR ("deoxyguanosine"[MeSH Terms] OR "deoxyguanosine"[All Fields] OR "deoxyguanosines"[All Fields]) OR ("4 hydroxy 2 nonenal"[Supplementary Concept] OR "4 hydroxy 2 nonenal"[All Fields] OR "4 hydroxy 2 nonenal"[All Fields]) OR "4-HNE"[All Fields] OR "hydroxynonenal"[All Fields] OR ("isoprostanes"[MeSH Terms] OR "isoprostanes"[All Fields] OR "isoprostane"[All Fields]) OR ("geodia"[MeSH Terms] OR "geodia"[All Fields] OR "isops"[All Fields]) OR (("superoxid"[All Fields] OR "superoxidant"[All Fields] OR "superoxides"[MeSH Terms] OR "superoxides"[All Fields] OR "superoxide"[All Fields]) AND ("dismutase"[All Fields] OR "dismutases"[All Fields])) OR "SOD"[All Fields] OR ("glutathione peroxidase"[MeSH Terms] OR ("glutathione"[All Fields] AND "peroxidase"[All Fields]) OR "glutathione peroxidase"[All Fields]) OR "GPx"[All Fields] OR (("mitofusin"[All Fields] OR "mitofusins"[All Fields]) AND "2"[All Fields]) OR ("Mtf"[All Fields] AND "2"[All Fields]))

WEB OF SCIENCE:

ALL FIELDS: (("POP" OR "Pelvic organ prolapse" OR "prolapse" OR "vaginal vault prolapse" OR "Descensus uteri" OR "metroptosis" OR "pelvic floor defect" OR "cystocoele" OR "cystocele" OR "rectocoele" OR "rectocele" OR "uretrocele" OR "anterior compartment defect" OR "middle compartment defect" OR "posterior compartment defect" OR "genitourinary prolapse") AND ("Oxidative stress" OR "oxidative damage" OR "reactive oxygen species" OR "ROS" OR "Advanced glycation endproducts" OR "8-hydroxydeoxyguanosine" OR "8-oxodG" OR "deoxyguanosine" OR "4-hydroxy-2-nonenal" OR "4-HNE" OR "hydroxynonenal" OR "isoprostane" OR "IsoPs" OR "superoxid dismutase" OR "SOD" OR "glutation peroxidase" OR "GPx" OR "Mitofusin 2" OR "Mtf 2"))

SCOPUS:

TITLE-ABS-KEY (("POP" OR "Pelvic organ prolapse" OR "prolapse" OR "vaginal vault prolapse" OR "Descensus uteri" OR "metroptosis" OR "pelvic floor defect" OR "cystocoele" OR "cystocele" OR "rectocoele" OR "rectocele" OR "uretrocele" OR "anterior compartment defect" OR "middle compartment defect" OR "posterior compartment defect" OR "genitourinary prolapse") AND ("Oxidative stress" OR "oxidative damage" OR "reactive oxygen species" OR "ROS" OR "Advanced glycation endproducts" OR "8-hydroxydeoxyguanosine" OR "8-oxodG" OR "deoxyguanosine" OR "4-hydroxy-2-nonenal" OR "4-HNE" OR "hydroxynonenal" OR "isoprostane" OR "IsoPs" OR "superoxid dismutase" OR "SOD" OR "glutation peroxidase" OR "GPx" OR "Mitofusin 2" OR "Mtf 2"))
